# Nanostructured PbSe Films Deposited by Spray Pyrolysis Using PbSe Colloidal Solutions

**DOI:** 10.3390/nano13182595

**Published:** 2023-09-20

**Authors:** Esteban Díaz-Torres, Jorge Roque, Alma Sofía Arreola-Pina, Manuel Alejandro Pérez-Guzmán, Rebeca Ortega-Amaya, Mauricio Ortega-López

**Affiliations:** 1 Sección de Electrónica del Estado Sólido, Departamento de Ingeniería Eléctrica, Centro de Investigación y de Estudios Avanzados del Instituto Politécnico Nacional, Av. IPN 2508, Ciudad de México 07360, Mexico; ediaz@cinvestav.mx (E.D.-T.); aarreola@cinvestav.mx (A.S.A.-P.); 2 Laboratorio Avanzado de Nanoscopía Electrónica, Centro de Investigación y de Estudios Avanzados del Instituto Politécnico Nacional, Av. IPN 2508, Ciudad de México 07360, Mexico; 3 Departamento de Física, Universidad Autónoma Metropolitana-Iztapalapa, Av. San Rafael Atlixco 186, Ciudad de México 09340, Mexico; pgmalejandro@gmail.com (M.A.P.-G.); orebeca@ymail.com (R.O.-A.); 4 CICFIM-Facultad de Ciencias Físico Matemáticas, Universidad Autónoma de Nuevo León, Av. Universidad S/N, Cuidad Universitaria, San Nicolás de los Garza 66451, Mexico

**Keywords:** lead selenide, nanostructures, polyol, amine, thiol, spray pyrolysis

## Abstract

This work describes the spray pyrolysis deposition of PbSe films, using as-prepared PbSe colloids as the starting solution. The PbSe colloids were prepared by using the alkahest approach, where Pb and Se precursors were made to react with the following green polyols: glycerin, ethylene glycol, and propylene glycol, to subsequently spray them onto glass substrates. The results of the characterization indicated that amine or thiol groups-free and single-phase rock-salt cubic PbSe powder was obtained, producing nanocrystals 16–30 nm in size. X-ray diffraction also showed that the PbSe films containing PbSeO_3_ and PbO·xH_2_O as impurity phases were produced during the deposition. The morphology of the powders and films was developed by a self-assembly process, in which the primary PbSe nanoparticles self-assemble to produce peanut-like microstructures. Additionally, a non-continuous and porous feature was formed in the thick films. Certain films revealed optical structures characterized by broad- and low-intensity bands resembling an exciton-like behavior. This could be attributed to the presence of nanocrystals with a size less than the Bohr radius, indicating reminiscent quantum effects. The results suggest that the usage of colloidal dispersions as spray solutions represents an effective approach to forming PbSe films, as well as that the synthesis method allows for the elimination of thiol and amine groups before deposition, significantly simplifying the process.

## 1. Introduction

Semiconductor nanocrystals have gained increasing attention in recent years due to their unique properties and potential applications in various fields. The nanocrystals’ optical and electrical properties strongly depend on their dimensions (size), shape, and surface functionality [1]. This singular dependence originates from the electron–hole pair interaction (or exciton) confined inside the minute volume of a nanocrystal. The Bohr exciton radius characterizes the spatial extent of an exciton in a nanocrystal. It defines the average distance between an electron and a hole bounded by Coulombic interaction. The Bohr exciton radius depends on several factors, including the material properties, dielectric constant, electron and hole effective masses, and the bandgap energy. It also relies on the nanocrystal size and shape, as these factors determine the spatial confinement of electrons and holes [2,3,4].

In addition to their outstanding physical properties, nanocrystals have also played a significant role in low-cost solution-based technologies. Appropriately sized and functionalized nanoparticles can easily be incorporated into suitable solvents to formulate ink-like dispersions. These dispersions have revolutionized the deposition of thin films by means of cost-effective methods, such as printing, spin-coating, and spray-coating techniques [5].

In this context, lead selenide (PbSe) has emerged as a promising candidate for developing novel optoelectronic and photoelectric devices [6,7,8,9,10,11], as it promises strong quantum confinement due to its narrow bandgap of 0.28 eV at 300 K, large Bohr exciton radius of 46 nm, and relative permittivity of 22.9. Therefore, the narrow bandgap of PbSe can be tuned by varying the nanocrystal size, allowing it to cover a significant energy range. These nanocrystals have the remarkable ability to absorb light across a wide spectrum of wavelengths, making them ideal for use in photovoltaic cells, photodetectors, and other optoelectronic devices. In the photovoltaic area, nanosized PbSe is intensively researched for quantum dots-based solar cells of wide spectral region [2,12,13], as theoretical studies have predicted that nanocrystalline PbSe can generate multiple electron–hole pairs (excitons) from a single absorbed photon (multiple exciton generation effect) [14,15]. Further PbSe applications include thermoelectric conversion [16,17], lasers [18], and IR spectrum detectors [19].

Nanocrystalline PbSe and thin films have been synthesized and deposited, respectively, by several techniques. The PbSe nanocrystal synthesis can be achieved by various methods, such as ball-milling (with high yield, but low control in composition, size, and shape) [20], co-precipitation (with high yield, scalable, but low control in composition, size, and shape) [21], solvothermal method (tunable size, shape, and properties, having issues with security, and long processing times) [22] and hot-injection (tunable size, shape, and properties, but low scalability and reproducibility) [23,24,25]. Chemical baths and electrochemical depositions are the most used methods for dense PbSe thin film production [26,27]. These methods are easy to implement in any laboratory because they use simple equipment, require low temperatures, and are low cost. To our best knowledge, there are two reports about the PbSe thin film deposition using colloidal dispersions or inks [12,28].

The use of polyols as a reaction medium is another widely used method to produce a variety of inorganic nanocrystalline systems [29,30,31]. Polyols are a kind of alcohols used as solvents and reducing agents in the colloidal synthesis of nanoparticles. They are preferred due to their low toxicity, high boiling points, and the ability to solubilize metal cations effectively. Other methods are the alkahest or universal solvent, where the amine–thiol mixture has led to the formation and deposition of nanocrystalline systems, mainly chalcogenides obtained by the direct dissolution of their elemental precursors [32,33].

In this work, we report our early studies on the nanocrystal PbSe synthesis and their simple deposition as a film by the spray pyrolysis technique, using combined methods of alkahest and polyols. We would like to emphasize that we have no knowledge about neither any studies combining both methods nor using different thiols and amines to those typically reported. Herein, green polyols (glycerin, ethylene glycol, and propylene glycol) and less corrosive amines (ethanolamine and triethanolamine) were used. The powders from the colloidal dispersion and the films were characterized structurally, morphologically, and optically.

## 2. Materials and Methods

### 2.1. PbSe Colloid Synthesis Procedure

Lead selenide (PbSe) was synthesized by the alkahest method (thiol–amine mixture) [32] in a polyol medium, using selenium powder (Se, 99.5% purity), ethanolamine (ETA, 98% purity), triethanolamine (TEA, 98% purity), thioglycolic acid (TGA, 98% purity), polyvinylpyrrolidone (PVP), lead nitrate (Pb(NO_3_)_2_, 99% purity), 1,2,3-propanetriol (glycerin, 99% purity), ethylene glycol (EG, 99.8% purity), and propylene glycol (PG, 99.5% purity). All the reactants were purchased from Sigma-Aldrich and used without further purification.

The lead precursor was prepared by dissolving 1.5 mmol of Pb(NO_3_)_2_ in 10 mL of some of the polyols listed above and heated up to 80 °C. The selenium precursor was prepared by mixing 1.5 mmol of selenium powder and 1.35 mL of some of the amines listed above under magnetic stirring. After 5 min, 0.15 mL of TGA was added, followed by 0.1 g of PVP, and 5 min later 20 mL of the selected polyol was added to disperse. This solution was heated up to 120 °C and preserved until the synthesis reaction was completed. The Pb and Se precursor solutions were mixed, and the final solution then was heated up to 180 °C for 1 h under magnetic stirring. Finally, the colloidal PbSe dispersion was cooled to room temperature for its processing as film. PbSe powders were recovered by centrifuging, decanting, and washing with ethanol six times and then evaporating ethanol in an oven at 60 °C overnight.

### 2.2. PbSe Film Deposition Procedure

**Figure 1:**
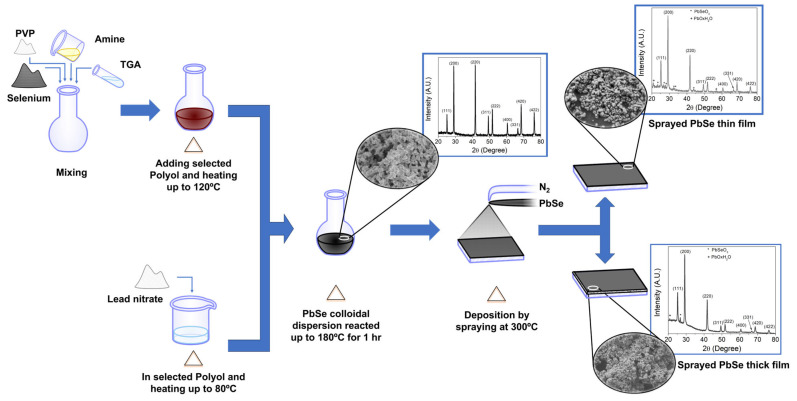
Schematic representation of PbSe colloidal synthesis and deposition procedure as a film by spray pyrolysis.

The nanostructured PbSe films were deposited using the spray pyrolysis technique. The deposition solutions were prepared by mixing two different volumes of the PbSe colloid (1 mL or 5 mL) with ethanol and 10 mL of deionized water up to 30 mL of total volume. Corning glass was used as a substrate, which was treated in piranha solution under sonication and dried in an oven at 80 °C. Deposition conditions were a substrate temperature of 300 °C and nitrogen flow as a carrier gas (sprayer). Thin and thick films were deposited by using 1 and 5 mL of colloidal PbSe, respectively. Colloids and films were labeled as glycerin-ETA, glycerin-TEA, EG-TEA, and PG-TEA, films are referred to as either thin or thick, and the synthesis conditions are summarized in Table 1. In summary, the synthesis and deposition methods described in Section 2.1 and Section 2.2 are outlined in Figure 1.

### 2.3. Sample Characterization

The obtained PbSe films were characterized structurally by X-ray diffraction (XRD), using a PANalytical XPERT-PRO diffractometer with Cu-Kα emission line, in the scan range of from 20° to 80° and a step 0.04°. Optical characterization was performed by spectral absorption measurements, using a JASCO V-670 spectrophotometer in the 400–1800 nm wavelength range with a resolution of 1.0 nm and by FTIR with a Thermo Nicolet Nexus 470 spectrometer in absorption mode and scan resolution of 2.0 cm^−1^. For FTIR characterization, PbSe powders and KBr were mixed to produce a pellet with a ratio of 1/50, PbSe/KBr W/W. Also, the powder and films were characterized morphologically by scanning electron microscopy (SEM), where a FE-SEM Zeiss Auriga 39–16 operating at between 1 and 2 kV was used.

## 3. Results and Discussion

### 3.1. Structural Properties

**Figure 2:**
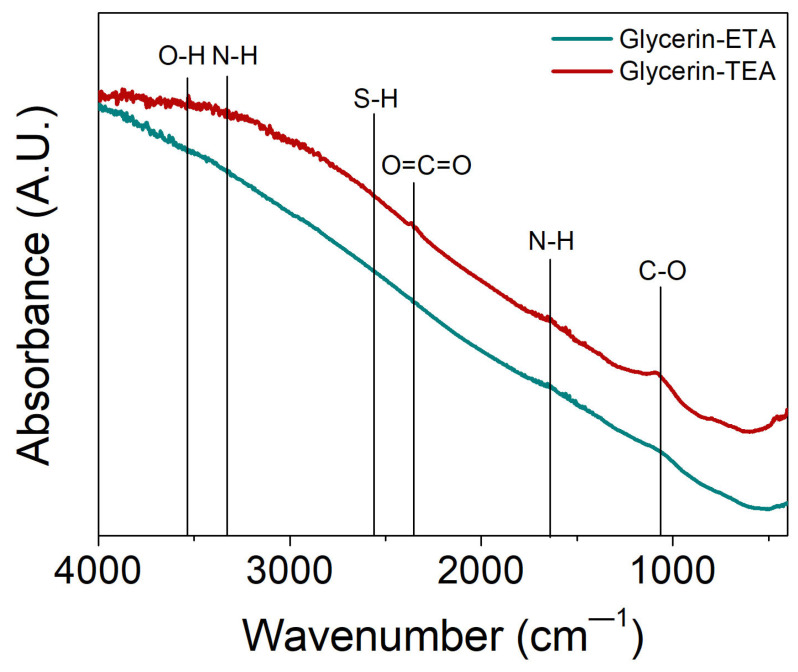
FTIR spectra for PbSe powders synthesized with ethanolamine (ETA) and triethanolamine (TEA) in glycerin. Vertical lines are used to identify the absorption bands of the main functional groups involved in the synthesis (amine and thiol).

The FTIR spectra of the PbSe powders recovered from the colloids synthesized using ETA and TEA are shown in Figure 2. In both cases, it is evident that the amine groups associated with the primary (ETA) and tertiary (TEA) amines, as well as the thiol group, are absent in PbSe, since their corresponding absorption bands were not identified around 3350, 1650, and 2550 cm^−1^, respectively. The lack of amines and thiols in our samples can be attributed to the temperature at which the synthesis process takes place. Specifically, when the temperature reaches 180 °C, the synthesis reaction is complete, hence both the amine and the thiol decompose into volatile byproducts. It is worth mentioning that our method allows for the elimination of amine and thiol from colloidal dispersion, instead of eliminating them during the deposition procedure, as it usually occurs and is reported by Weeber et al. [34]. The elimination of the amine and thiol groups is critical, because their presence could act as surface impurities in PbSe, constituting physical barriers that affect the electric charge transport in real-world applications.

**Figure 3:**
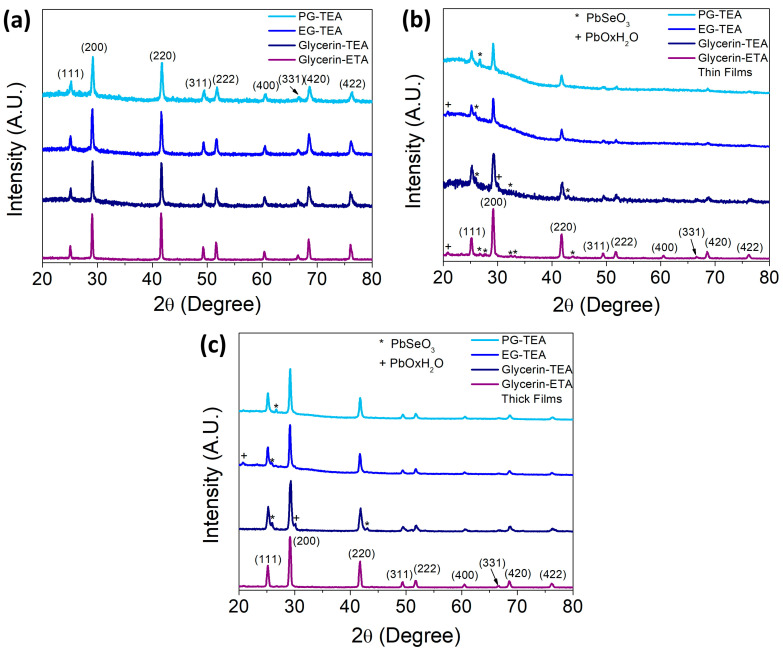
XRD patterns of (**a**) PbSe powders recovered from the colloidal dispersion, (**b**) PbSe thin films, and (**c**) PbSe thick films. Cross and star marks are used to indicate secondary phases in the films.

Figure 3a displays the corresponding X-ray diffraction pattern of PbSe powders. In general, well-crystallized powders were obtained and non-other impurity phases were detected. All observed peaks on the XRD patterns could be indexed to the PbSe rock-salt face-centered cubic structure (ICDD 00-006-035 card). It was found that the lattice parameter (*a*) ranged from 6.113 Å to 6.132 Å, indicating that the PbSe crystalline lattice was slightly distorted relative to its reference value of 6.121 Å. The lattice parameter was calculated from the average of all diffraction peaks. A compressive strain of 0.13% was observed in powders synthesized using propylene glycol and TEA, whereas a tensile strain of 0.18% was obtained for powders prepared using glycerin and ETA. The crystal size (δ) was estimated from the Scherrer equation, considering spherical crystals, *k* = 0.89, and the full width at half maximum (FWHM) of the most intense diffraction peaks, which are (111), (200), and (220). In a similar way to the lattice parameter, the crystal size decreases when propylene glycol and TEA are used in the colloidal dispersion and is larger for the glycerin-ETA system, see Table 2. The role of the tested polyols as a dispersant in the colloids is clear; glycerin maintains a weak dispersion during the reaction, allowing the ripening and growth of nanocrystals. In contrast, ethylene glycol and propylene glycol allow for greater dispersion and limit the nanocrystal growth. In addition, in any case, the polyols maintain the PbSe colloids stable, preventing their oxidation.

The X-ray diffraction patterns of the sprayed films are presented in Figure 3b,c for thin and thick films, respectively. Both films are predominantly composed of rock-salt PbSe, as indicated by the high-intensity diffraction peaks. However, unlike the powdered single-phase PbSe, thin and thick films contain impurity phases of PbSeO_3_ and PbO·xH_2_O. The low intensity of their diffraction peaks suggests that these impurity phases are present only in trace amounts. The precise origin of impurity phases that exclusively form on sprayed films poses a significant challenge. Nevertheless, it is reasonable to assume that these phases arise from the oxidation of PbSe during the spraying process, considering that the reactivity of nanocrystals is heightened as their size decreases. Furthermore, the oxidation phenomenon becomes more pronounced in thin films, where smaller PbSe crystals are deposited, in contrast to those in thick films where only a small quantity of nanocrystals smaller than those of average sizes (Table 2) is transformed into the identified phases. 

It is worth noting that all the films consist of PbSe nanocrystals that are smaller than those found in the powder; for example, the thin films are composed of the smallest PbSe nanocrystals. This finding is remarkable as it suggests that the mechanism of nanocrystal coalescence differs significantly between the powder and thin films. Nanocrystal coalescence begins once the polyol is removed, and this process continues as the nanocrystals are manipulated to produce either powders or films. Considering the anticipated increase in coalescence with the colloid concentration, it is reasonable to infer that this factor contributes to the larger crystal sizes observed in thick films, in contrast to their thin film counterparts.

In all instances, the calculated lattice parameter resulted smaller than the reference value, indicating that the cell is compressed both in thin and thick films; the most compressed film (0.30%) was that thick film whose colloid contained glycerin and TEA. The crystal size depends on whether the film is thin or thick, thin films are constituted by small nanocrystals ranging from 9.33 to 20.52 nm, whereas thick films have larger size nanocrystals from 17.94 to 22.26 nm.

### 3.2. Microstructure

**Figure 4:**
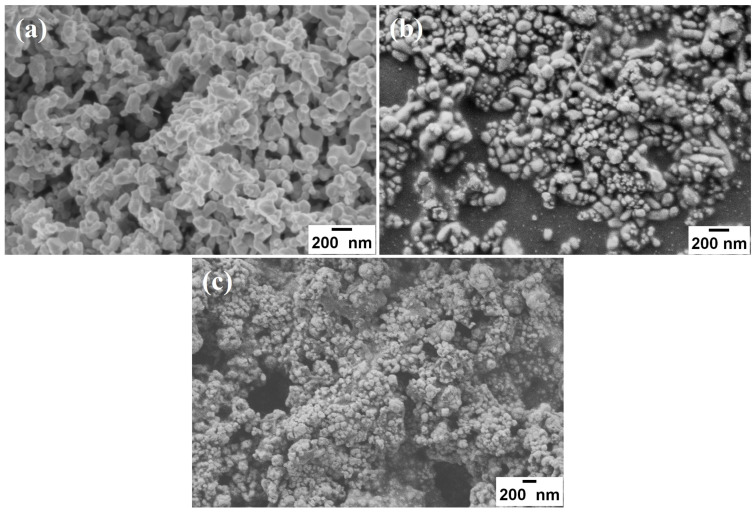
SEM images representative for all experimental conditions assayed and corresponding to glycerin-ETA system. (**a**) PbSe powder recovered from the colloidal dispersion, (**b**) PbSe thin film, and (**c**) PbSe thick film.

The SEM images of representative samples are shown in Figure 4 and correspond to the glycerin-ETA synthesized ones. The morphology of both powders and films considerably differs from each other. The images suggest that major morphological features originate from a combination of coalescence and self-assembly mechanisms, as evidenced in a previous report [21]. In the case of PbSe powder (Figure 4a), their morphology seems to have developed by sequential self-assembly; from PbSe nanoparticles 30 nm in size, to peanut-like microstructures. 

When PbSe colloids are deposited by spray pyrolysis, the resulting film morphology is clearly dependent on the deposition conditions, and the mechanisms of self-assembly and coalescence also appear to be involved. By varying the colloidal concentration in the deposition solution, from diluted to concentrated, the structures developed by nanocrystal agglomeration increase in size, ranging from thin to thick films (Figure 4b,c). Thus, diluted solutions produce thin films with small and dispersed nanocrystals, whereas concentrated solutions lead to thick and porous films with a high density of agglomerated nanocrystals. Larger crystals are formed during the deposition process by coalescence and self-assembly of nanocrystals whose size distribution is centered in the values reported in Table 2. Nanocrystal agglomeration is likely causing the compression stress calculated from the X-ray diffractograms of the films. 

Spray pyrolysis is a cost-effective and widely used technique for depositing polycrystalline thin films on various substrates. The conventional version of this technique uses a liquid solution containing the compound of interest or the dissolved precursors. A carrier gas is then used to drive this deposition solution by means of a spray nozzle, which atomizes the solution and directs it toward the heated substrate surface. At the surface, precursors react chemically to form the desired solid compound, while the solvent and byproducts volatilize far away from the substrate [35]. 

If colloids are used as the spraying solution, it is crucial for the solvent to evaporate prior to reaching the hot substrate surface. This allows the dried particles to self-organize and coalesce, ultimately forming a uniform and well-compacted film. Unfortunately, it seems that these conditions were not met in our experiments because non-continuous and uneven surface PbSe films were obtained; this observation is supported by the SEM characterization. In addition, the agglomerated nanocrystals within the films exhibit a porous structure, rendering them unsuitable for application in film-based devices [2,6,12].

### 3.3. Optical Properties

**Figure 5:**
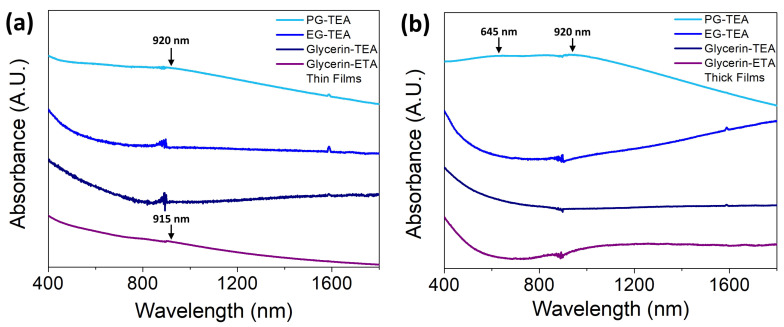
Optical absorption spectra of (**a**) PbSe thin films and (**b**) PbSe thick films in the UV-Vis-near IR region. Arrows were added to indicate the location of the excitonic-like bands in the spectra.

Figure 5a,b displays the absorption spectra of thin and thick films, respectively. All deposited PbSe films display significant absorption in the 400–1800 nm wavelength range. The thin films processed with glycerin-ETA and propylene glycol-TEA colloids display a low intensity and broad absorption band similar to that of excitons, arising between 900 nm and 950 nm. Films processed with glycerin-TEA and ethylene glycol-TEA colloids show broadband from 825 nm and extending beyond 1800 nm in wavelength. Figure 5b displays the absorption spectra for each thick film. Here, the PbSe film only processed with the propylene glycol-TEA colloid exhibits an absorption band resembling exciton behavior. This absorption band results from the overlapping of two individual lines located approximately at 645 nm and 920 nm. The rest of the thick films behave like their thin film counterparts processed with glycerin-TEA and ethylene glycol-TEA colloids. No excitonic absorption was evidenced in the films that absorb in a near-IR deeper region (broadband from ~700 nm and extending beyond 1800 nm). Notice that thin and thick films exhibit strong absorption in the 400–900 nm wavelength range.

PbSe films have a polydisperse size distribution, since all absorption bands are broad, with an average size centered on the XRD values. However, those films displaying the 800–1800 nm band are likely constituted by a high density of nanocrystals of size δ and a second distribution of sizes larger than δ, as a result of the self-assembly process during the deposition. Under such conditions, the larger size distribution behaves as a single large crystal, which absorbs low-energy photons. For the films where excitonic-like absorption was shown, thin films (750–1200 nm) and the thick film (450–1200 nm), their nanocrystal distribution is wide and is only centered on δ since the nanocrystal average size is less than the Bohr exciton radius [36], which suggests that this nanocrystalline PbSe still exhibits quantum confinement. The singular case of the PG-TEA thick film, whose optical structure has two overlapped excitonic-like absorption bands at 645 nm and 920 nm. This can be attributed to the splitting in two energy levels near the conduction band with energies of 1.92 eV and 1.35 eV, respectively. This undoubtedly confirms the presence of quantum effects in that film [4]. Because of the limited optical characterization, it is challenging to determine the exact origin of the excitonic-like bands observed in those films with such a feature; therefore, further in-depth investigation is necessary.

## 4. Conclusions

Colloidal PbSe was successfully synthesized by the alkahest approach, dissolving elemental selenium in various polyols (ethylene glycol, glycerin, and propylene glycol). As-synthesized PbSe colloids were used directly for the easy deposition of PbSe films by the spray pyrolysis technique. All synthesis routes assayed in this study, produced well-crystallized, single-phase PbSe nanocrystals, free from amines and thiols and with a size between 16 nm and 30 nm. In turn, as-deposited PbSe films exhibited nanocrystals ranging from 9 nm to 20 nm for thin films and from 18 to 22 nm for thick films. The films showed the presence of secondary phases, namely, PbSeO_3_ and PbO·xH_2_O. 

The morphological characteristics of both powders and films evolve in sequential steps. In powders, the primary nanoparticles coalesce and self-assemble to develop peanut-like microstructures. In the thin films, single, self-assembled, and coalesced nanocrystals were dispersed on the substrate. In contrast, the thick films showed clusters of single and self-assembled nanocrystals, agglomerated, resulting in non-continuous and porous films. This could mean a drawback for photovoltaic or switching applications, where even and well-compacted films are needed; nevertheless, these films may be suitable for applications that require a large surface area. Further research is necessary to optimize the deposition process and achieve more uniform and compact films. PbSe films with less polydisperse size distribution exhibited absorption resembling excitons due to reminiscent quantum effects. Colloids synthesized with propylene glycol-TEA, and their corresponding films, displayed the best properties and were the most similar to a nanocrystalline system.

## Figures and Tables

**Table 1:** Main synthesis parameters of PbSe nanocrystals produced at 180 °C for 1 h.

Label/Sample	Thiol	Amine	Solvent
Glycerin-ETA	TGA	ETA	Glycerin
Glycerin-TEA	TGA	TEA	Glycerin
EG-TEA	TGA	TEA	Ethylene glycol
PG-TEA	TGA	TEA	Propylene glycol

**Table 2:** Lattice parameter and crystal size for PbSe powders, thin and thick films.

Type	Powders		Thin Film		Thick Film	
Sample	*a* (Å)	δ (nm)	*a* (Å)	δ (nm)	*a* (Å)	δ (nm)
Glycerin-ETA	6.132	30.05	6.117	20.52	6.114	22.26
Glycerin-TEA	6.123	20.45	6.105	12.9	6.103	17.94
EG-TEA	6.124	18.42	6.112	9.33	6.115	19.53
PG-TEA	6.113	16.12	6.110	11.83	6.112	20.59

## Data Availability

Not applicable.
